# Transcatheter Mitral Edge-to-Edge Repair With Intra-Aortic Balloon Pump Support in Severe Left Ventricular Dysfunction

**DOI:** 10.1016/j.jaccas.2026.108870

**Published:** 2026-06-12

**Authors:** Sary Wedinly, Rasha Al-Bawardy, Wail Kashkari, Abdullah Alsaiedi, Ahmed Krimly, Saad Albugami, Ibrahim Jelaidan, Malek Merdad, José Andres Fernandez, Anas Merdad

**Affiliations:** a King Abdullah International Medical Research Center, Jeddah, Saudi Arabia; b King Faisal Cardiac Center, King Abdulaziz Medical City, Ministry of National Guard Health Affairs, Jeddah, Saudi Arabia; c College of Medicine King Saud Bin Abdulaziz University for Health Sciences, Jeddah, Saudi Arabia; d Faculty of Medicine, King Abdulaziz University, Jeddah, Saudi Arabia; e Department of Anesthesiology, King Faisal Cardiac Center, Ministry of National Guard Health Affairs, Jeddah, Saudi Arabia

**Keywords:** edge-to-edge repair, intra-aortic balloon pump (IABP), severe left ventricular dysfunction, transcatheter mitral repair

## Abstract

**Background:**

Severe functional mitral regurgitation (MR) with advanced left ventricular (LV) dysfunction carries poor outcomes despite guideline-directed medical therapy. Patients with LV ejection fraction ≤20%, severely dilated ventricles, or hemodynamic instability were excluded from landmark trials.

**Case Summary:**

Five patients (LV ejection fraction: 9%-29%) with severe functional MR, ineligible for transplantation or LV assist device, underwent transcatheter edge-to-edge repair (TEER) with intra-aortic balloon pump (IABP) support. Procedural success was achieved in all, including 1 patient after a second TEER for recurrent MR.

**Why Beyond the Guidelines:**

These patients fall outside COAPT (Cardiovascular Outcomes Assessment of the MitraClip Percutaneous Therapy for Heart Failure Patients With Functional Mitral Regurgitation) and current valvular guidelines, which offer no periprocedural mechanical support strategy.

**Discussion:**

IABP provided hemodynamic stability and may have mitigated afterload mismatch. All patients achieved NYHA functional class I-II status at discharge, sustained on follow-up.

**Take-Home Message:**

Prophylactic IABP might enable safe TEER in patients with LV ejection fraction ≤20% and severe functional MR who fall outside landmark trial criteria; a heart team approach may extend TEER to advanced heart failure patients with no other options.

## Visual Summary

**Figure:**
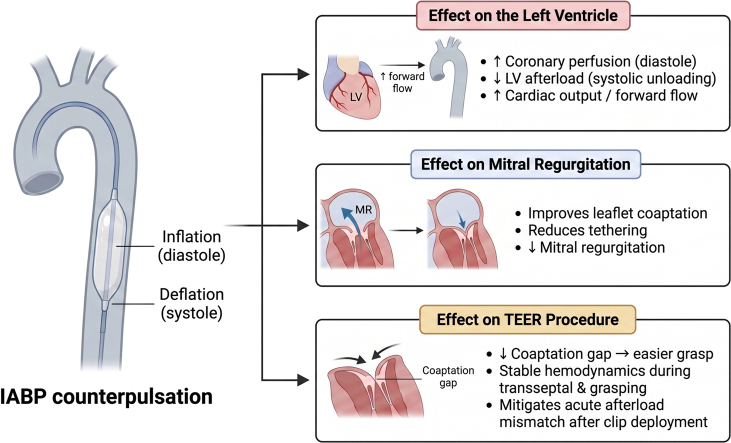

**Visual Summary:**
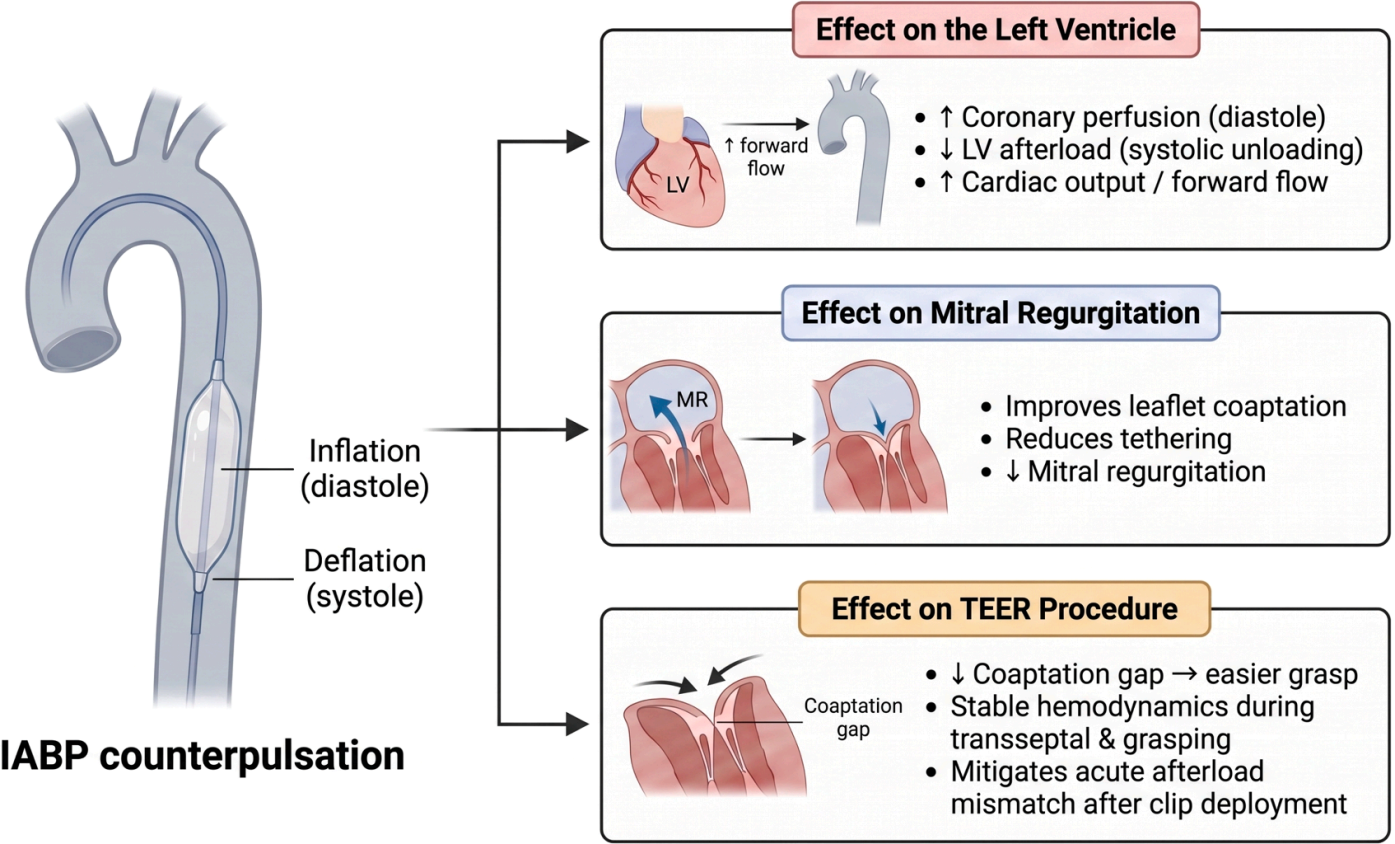
Mechanistic Rationale for Intra-Aortic Balloon Pump Support During Transcatheter Edge-to-Edge Mitral Repair in Patients With Severe Left Ventricular Dysfunction IABP counterpulsation, with inflation in diastole and deflation in systole, exerts 3 synergistic effects that support TEER in patients with severe LV dysfunction and severe secondary mitral regurgitation. (Top Right) Effect on the left ventricle: Diastolic inflation augments coronary perfusion, while systolic deflation reduces LV afterload, resulting in increased cardiac output and forward flow. (Middle Right) Effect on mitral regurgitation: Afterload reduction and improved LV geometry enhance leaflet coaptation and reduce papillary muscle tethering, leading to a decrease in regurgitant volume. (Bottom Right) Effect on the TEER procedure: A narrower coaptation gap facilitates leaflet grasping, hemodynamic stability is maintained during transseptal puncture and device manipulation, and the IABP mitigates acute afterload mismatch after clip deployment. Together, these effects provide a physiologic safeguard that may extend TEER to high-risk patients with profound LV dysfunction who fall outside conventional trial eligibility. IABP = intra-aortic balloon pump; LV = left ventricle; MR = mitral regurgitation; TEER = transcatheter edge-to-edge repair.

Transcatheter edge-to-edge repair (TEER) improves mortality, heart failure hospitalizations, and quality of life in patients with significant functional mitral regurgitation (MR) and is now a pillar of heart failure management.^1^ However, periprocedural management of advanced heart failure, characterized by low cardiac output, medication intolerance, and hypotension, is hemodynamically challenging, particularly during induction. The landmark COAPT (Cardiovascular Outcomes Assessment of the MitraClip Percutaneous Therapy for Heart Failure Patients With Functional Mitral Regurgitation) trial excluded patients with left ventricular ejection fraction (LVEF) <20%, hemodynamic instability, or need for inotropic or mechanical circulatory support, leaving a substantial evidence gap for this high-risk population.^2^

An intra-aortic balloon pump (IABP) may serve 2 complementary roles during mitral TEER. It provides hemodynamic support during induction and the postprocedural period, when ejection fraction may transiently decline after reduction of mitral regurgitation. Emerging evidence also suggests that an IABP can improve mitral coaptation by reducing the leaflet gap, facilitating clip deployment in patients with large coaptation gaps.^3^ In addition, limited data have demonstrated its feasibility and safety in patients with cardiogenic shock.^4^

We describe the feasibility and short-term outcomes of mitral TEER with IABP support in 5 patients with profound left ventricular (LV) dysfunction and symptomatic severe functional MR who fall outside trial-eligible populations.

## Case Summaries

### Case 1

#### Patient information

A 77-year-old man with a history of hypertension, nonischemic cardiomyopathy, severe functional MR, atrial fibrillation, and chronic kidney disease stage 2 (estimated glomerular filtration rate [eGFR]: 48 mL/min) presented with NYHA functional class II dyspnea despite maximum tolerated guideline-directed medical therapy (GDMT).

#### Investigations

Transthoracic echocardiography (TTE) showed an LVEF of 29% (Simpson's biplane), LV end-diastolic indexed volume (LVEDVi) of 83 mL/m^2^, severe functional MR, and right ventricular systolic pressure (RVSP) of 69 mm Hg. B-type natriuretic peptide (BNP) was 2,854 pg/mL (Table 1).

**Table 1:** Patient Characteristics, Procedural Details, and Outcomes of Patients Undergoing TEER With IABP Support

	Case 1	Case 2	Case 3	Case 4	Case 5
Baseline patient characteristics					
Age	77	75	65	58	31
Sex	Male	Male	Male	Female	Male
Hypertension	Yes	Yes	No	Yes	No
Diabetes	No	Yes	Yes	No	No
Dyslipidemia	No	Yes	Yes	Yes	No
Atrial fibrillation	Yes	Yes	Yes	No	No
Obstructive CAD	No	Yes	Yes	No	No
Prior PCI	No	Yes	Yes	No	No
Prior CABG	No	No	No	No	No
Prior stroke	No	No	No	No	No
Peripheral vascular disease	No	No	No	No	No
STS risk score for MV repair (%)	3.6	9.9	3.9	12.6	3.25
EuroSCORE II risk score for non-CABG 1 procedure (%)	6.4	7.9	3.18	4.6	3.88
NYHA functional class preprocedure	II	III	III	II	III
Hemoglobin (g/dL)	15	13.5	16.8	12	12
eGFR (mL/min)	48	42	90	6	31
Creatinine clearance (mL/min)	50	35	110	7	27
BP (mm Hg)	120/51	92/76	106/77	113/72	111/73
LVEF (%)	29	9	10	19	11
LVEDVi (mL/m^2^)	83	92	153	119	207
LVEDD (mm)	58	64	73	69	74
TAPSE (mm)	17	14	14	18	17
EROA (cm^2^)	0.52	0.50	0.59	0.48	0.35
MR severity	+4	+4	+4	+4	+4
Etiology of MR	Functional	Functional	Functional	Functional	Functional
Procedural details					
Procedural success	Yes	Yes	Yes	Yes	Yes (after 2nd repair)
Number of clips	1	1	1	1	2
Size of clips	XTW	XTW	P10	P10	XTW, XT
Post mean gradient (mm Hg)	3	2	3	4	5
IABP duration (days)	1	1	2	1	1
IABP withdrawal in catheterization laboratory	Yes	Yes	No	Yes	Yes
Patient outcomes					
MR severity at discharge	+1	+1	+1	+2	+2
NYHA functional class at discharge	I	I	I	I	II
Length of ICU stay	1	4	4	10	5
Length of stay (days)	10	7	5	10	6
Death on admission	No	No	No	No	No
Death at last follow-up	No	No	No	No	No
IABP indication	Persistent hypotension after anesthesia induction	Prophylactic given severe LV dysfunction	Prophylactic given severe LV dysfunction	Prophylactic given severe LV dysfunction	Prophylactic given severe LV dysfunction

AF = atrial fibrillation; BP = blood pressure; CABG = coronary artery bypass grafting; CAD = coronary artery disease; eGFR = estimated glomerular filtration rate; EROA = effective regurgitant orifice area; IABP = intra-aortic balloon pump; ICU = intensive care unit; LVEDD = left ventricular end-diastolic diameter; LVEDVi = indexed left ventricular end-diastolic volume; LVEF = left ventricular ejection fraction; MR = mitral regurgitation; MV = mitral valve; PCI = percutaneous coronary intervention; STS = Society of Thoracic Surgeons; TAPSE = tricuspid annular plane systolic excursion; TEER = transcatheter edge-to-edge repair.

#### Interventions

The patient developed hypotension after induction; an IABP was inserted as a rescue measure at 1:1 augmentation. A single MitraClip XTW (Abbott) was implanted successfully, with residual mild MR. The IABP was removed at the end of the procedure without complications.

#### Follow-up

At discharge, the patient had mild residual MR and was at NYHA functional class I status. At 6 months, he was at NYHA functional class II status with an LVEF of 28% and mild to moderate MR.

### Case 2

#### Patient information

A 75-year-old man with a history of hypertension, ischemic cardiomyopathy, severe functional MR, atrial fibrillation, and chronic kidney disease stage 3a (eGFR 42 mL/min) presented with persistent NYHA funcational class III dyspnea despite high-dose diuretics and maximum tolerated GDMT.

#### Investigations

TTE showed an LVEF of 9%, LVEDVi of 92 mL/m^2^, severe functional MR, and RVSP of 40 mm Hg. BNP was 3,245 pg/mL (Table 1).

#### Interventions

A prophylactic IABP was inserted at 1:1 augmentation before TEER. A single MitraClip XTW was implanted successfully, with mild residual MR. The IABP was removed at the end of the procedure without complications.

#### Follow-up

At discharge, he had mild residual MR and was considered NYHA functional class I status. At 3 months, he remained at NYHA functional class I status, with an LVEF of 20% and mild to moderate MR.

### Case 3

#### Patient information

A 65-year-old man with a history of diabetes mellitus, coronary artery disease, ischemic cardiomyopathy, severe functional MR, and obesity presented with NYHA functional class III dyspnea, limiting daily activities.

#### Investigations

TTE showed an LVEF of 10%, LVEDVi of 153 mL/m^2^, severe functional MR, and RVSP of 55 mm Hg. BNP was 152 pg/mL (Table 1).

#### Interventions

A prophylactic IABP was inserted at 1:1 augmentation before TEER. A single MitraClip was implanted successfully, with residual mild to moderate MR. The IABP was removed the following day without complications.

#### Follow-up

At discharge, there was moderate residual MR, and he was considered at NYHA functional class I status. At 2 years, he remained at NYHA functional class I status, with an LVEF of 20%, moderate MR, and no heart failure hospitalizations.

### Case 4

#### Patient information

A 58-year-old woman with a history of hypertension, coronary artery disease, advanced heart failure, severe functional MR, and advanced chronic kidney disease (eGFR: 6 mL/min) presented with NYHA functional class II dyspnea despite maximum tolerated GDMT.

#### Investigations

TTE showed an LVEF of 19%, severely dilated LV (LVEDVi: 119 mL/m^2^), severe functional MR, and RVSP of 79 mm Hg. BNP was 4,902 pg/mL (Table 1).

#### Interventions

A prophylactic IABP was inserted at 1:1 augmentation before TEER. A single MitraClip was implanted successfully, with residual moderate MR. The IABP was removed after the procedure in the catheterization laboratory without complications.

#### Follow-up

At discharge, the patient had moderate residual MR and was at NYHA functional class I status. At 8 months, she remained at NYHA functional class I status, with no heart failure hospitalizations.

### Case 5

#### Patient information

A 31-year-old man had a history of advanced heart failure with biventricular failure, severe functional MR, severe functional tricuspid regurgitation, and chronic kidney disease stage 4 (eGFR: 31 mL/min). He was not a candidate for heart transplantation and refused an LV assist device. He presented with NYHA functional class III dyspnea and recurrent heart failure admissions, and remained symptomatic despite maximum tolerated GDMT and high-dose diuretics.

#### Investigations

TTE showed an LVEF of 11%, severely dilated LV (LVEDVi: 207 mL/m^2^), severe functional MR, and RVSP of 31.8 mm Hg. BNP was 11,489 pg/mL (Table 1).

#### Interventions

##### First procedure

A prophylactic IABP was inserted at 1:1 augmentation before TEER. A single MitraClip XTW was implanted successfully, with residual moderate MR; the procedure was halted at 1 clip to avoid afterload mismatch given profound LV dysfunction. The IABP was removed at the end of the procedure without complications. Follow-up echocardiography revealed recurrent moderate to severe MR, prompting a second procedure.

##### Second procedure

Repeat TEER under IABP support was performed; a second MitraClip XT was implanted, reducing MR to mild to moderate. The IABP was removed at the end of the procedure without complications.

##### Follow-up

The patient was at NYHA functional class II status at discharge after both the first and the second TEER. He was readmitted once with heart failure within 30 days after the procedure.

## Why Beyond the Guidelines

Of our 5 patients, 4 patients fell outside the eligibility criteria that underpin current guideline recommendations for TEER in secondary MR. The COAPT trial restricted enrollment to patients with an LVEF of 20% to 50%, LV end-diastolic diameter of ≤70 mm, pulmonary arterial systolic pressure of ≤70 mm Hg, and the absence of cardiogenic shock or inotrope dependence, criteria that continue to anchor the corresponding recommendations in current American College of Cardiology/American Heart Association and European Society of Cardiology/European Association for Cardio-Thoracic Surgery valvular heart disease guidelines.^1^^,^^2^

Among our cohort, 4 patients had an LVEF below the COAPT 20% threshold, 3 patients had an LV end-diastolic diameter beyond the 70-mm ceiling, 1 patient had a pulmonary arterial systolic pressure above 70 mm Hg, and 1 patient had biventricular failure. None were candidates for heart transplantation or durable LV assist device support, leaving TEER as their only mechanical option for MR reduction.

Current guidelines do not address whether or how prophylactic mechanical circulatory support should be used to enable TEER in this population. Our institutional approach of prophylactic or rescue IABP for periprocedural hemodynamic stabilization represents a pragmatic strategy that is neither endorsed nor proscribed by existing recommendations.

## Case Outcome and Follow-Up

Procedural success was achieved in all 5 patients, with no IABP-related complications (limb ischemia, bleeding, or thromboembolism). Case 5 required a second TEER for recurrent moderate to severe MR, performed again under IABP support, after which MR was reduced to mild to moderate. Residual MR at discharge ranged from mild to moderate, and all patients improved to NYHA functional class I-II status at discharge. One 30-day readmission occurred (case 5, right-heart failure). On longest available follow-up (3 months to 2 years), functional gains were sustained, with no further heart failure hospitalizations. Per-case outcomes are summarized in Table 1.

## Discussion

This series highlights the feasibility of mitral TEER with IABP support in patients with advanced heart failure and severe secondary MR outside COAPT eligibility.^1^ IABP was prophylactic in 4 cases and rescue in 1 case and was safely removed in all patients at the end of the procedure with no IABP-related complications.

Our findings align with prior evidence that mechanical circulatory support can mitigate periprocedural hemodynamic instability during TEER in vulnerable patients.^3^^,^^5^^,^^6^ Prophylactic IABP addresses this by stabilizing hemodynamics during induction and the postclip period when transient ejection fraction decline may occur.^7^^,^^8^

IABP augments cardiac output, reduces afterload, and enhances coronary perfusion—critical in patients prone to periprocedural hypotension or afterload mismatch after MR reduction.^6^^,^^7^ Prior reports support IABP-enabled TEER in patients with vulnerable hemodynamics;^3^^,^^5^^,^^6^ our cohort had more remodeled ventricles and lower ejection fraction than most published series, extending the utility of IABP to a nonshock, prohibitive-risk population.

Existing data on mechanical circulatory support during TEER are largely drawn from cardiogenic shock cohorts. A prospective registry of 30 cardiogenic shock patients with significant MR who underwent TEER under mandated mechanical circulatory support reported 83.3% procedural success and a 6-month survival rate of 73.3%.^9^ A meta-analysis of TEER in acute MR with cardiogenic shock reported 86.2% procedural success and lower in-hospital mortality than conservative management (odds ratio: 0.64).^10^ Multicenter reviews corroborate success rates of 85% to 94% in cardiogenic shock cohorts, with TEER serving as a bridge to advanced therapies.^5^^,^^11^ These findings suggest that prophylactic IABP, as employed in our series, may extend the benefits of TEER to “prohibitive-risk” patients beyond trial-eligible populations.

Limitations of this case series include the small, retrospective single-center design without a non-IABP comparator, which precludes causal inference. Follow-up duration varied (3 months to 2 years), and objective metrics such as serial echocardiography and quality-of-life scores were not uniformly available.

## Conclusions

Our real-world experience supports prophylactic or rescue IABP during TEER for patients with profound LV dysfunction and severe functional MR who fall outside guideline-directed eligibility. A multidisciplinary heart team approach with planned hemodynamic support may safely extend TEER to this population, and prospective studies are needed to define the optimal strategy.

## Funding Support and Author Disclosures

The authors have reported that they have no relationships relevant to the contents of this paper to disclose.

**Take-Home Message**

• Prophylactic intra-aortic balloon pump use might enable safe transcatheter edge-to-edge repair in patients with left ventricular ejection fraction ≤20% and severe functional mitral regurgitation who fall outside landmark trial criteria.
